# Targeted enrichment beyond the consensus coding DNA sequence exome reveals exons with higher variant densities

**DOI:** 10.1186/gb-2011-12-7-r68

**Published:** 2011-07-25

**Authors:** Matthew N Bainbridge, Min Wang, Yuanqing Wu, Irene Newsham, Donna M Muzny, John L Jefferies, Thomas J Albert, Daniel L Burgess, Richard A Gibbs

**Affiliations:** 1 Human Genome Sequencing Center, Baylor College of Medicine, One Baylor Plaza, Houston, TX 77030, USA; 2 Department of Structural and Computational Biology and Molecular Biophysics, Baylor College of Medicine, One Baylor Plaza, Houston, TX 77030, USA; 3 Department of Pediatrics-Cardiology, Baylor College of Medicine, One Baylor Plaza, Houston, TX 77030, USA; 4 Roche NimbleGen, Inc., 504 S. Rosa Road, Madison, WI 53719, USA

## Abstract

**Background:**

Enrichment of loci by DNA hybridization-capture, followed by high-throughput sequencing, is an important tool in modern genetics. Currently, the most common targets for enrichment are the protein coding exons represented by the consensus coding DNA sequence (CCDS). The CCDS, however, excludes many actual or computationally predicted coding exons present in other databases, such as RefSeq and Vega, and non-coding functional elements such as untranslated and regulatory regions. The number of variants per base pair (variant density) and our ability to interrogate regions outside of the CCDS regions is consequently less well understood.

**Results:**

We examine capture sequence data from outside of the CCDS regions and find that extremes of GC content that are present in different subregions of the genome can reduce the local capture sequence coverage to less than 50% relative to the CCDS. This effect is due to biases inherent in both the Illumina and SOLiD sequencing platforms that are exacerbated by the capture process. Interestingly, for two subregion types, microRNA and predicted exons, the capture process yields higher than expected coverage when compared to whole genome sequencing. Lastly, we examine the variation present in non-CCDS regions and find that predicted exons, as well as exonic regions specific to RefSeq and Vega, show much higher variant densities than the CCDS.

**Conclusions:**

We show that regions outside of the CCDS perform less efficiently in capture sequence experiments. Further, we show that the variant density in computationally predicted exons is more than 2.5-times higher than that observed in the CCDS.

## Background

Single nucleotide variants (SNVs) and short indels can be discovered by hybridization-based targeted enrichment, followed by high-throughput DNA sequencing. This 'capture sequencing' can target the protein coding regions of the genome, the 'exome', and provide a cost-effective alternative to whole genome sequencing (WGS) [1-6]. Capture sequencing has now been applied to the identification of pathogenic variants in several disease models [7-16] and in population studies comparing phenotypically normal individuals [17].

DNA may be enriched by a number of methods [1,4,5,18]. Here, we perform liquid-phase hybridization using biotinylated, DNA-oligonucleotide probes with a typical length of 60 to 80 bp. The probes are incubated with fragmented genomic DNA, after ligation with sequencing-platform specific adapters. Subsequently, the desired regions are recovered via streptavidin-coated magnetic beads with affinity for the biotinylated oligonucleotide probes. This approach has allowed interrogation of a human exome, beginning with as little as 1 μg of total DNA and with just 3 Gbp of total raw sequence [6].

The consensus coding DNA sequence (CCDS) [19] exons have been used most frequently to guide the design of capture reagents because their gene models are robust and encompass only approximately 30 Mbp. The CCDS gene collection, however, is defined by conservative criteria and lacks many of the genes found in other sets, such as RefSeq [20]. Even when two gene collections share the same core genes, the underlying exon content of those genes may vary. Consequently, the optimal design choice for targeting protein coding regions is a matter of ongoing concern.

In addition, many regions related to gene function, such as transcription factor binding sites, enhancer sites and UTRs, exist outside of the coding exome. These elements may also contribute to disease pathogenicity and are desirable components of target probe sets. Understanding the expected variant density of these regions and our ability to sequence them are therefore important considerations for designing future capture experiments.

**Figure 1:**
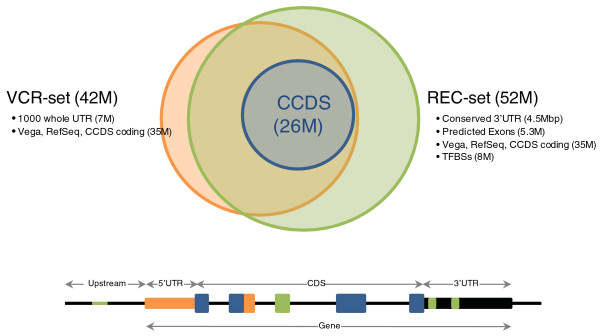
**Content and overlap of the VCR-set and REC-set designs**. A hypothetical gene shows regions that would be targeted by the VCR-set (orange), REC-set (green), both designs (orange/green) and CCDS (blue). TFBS, transcription factor binding site.

**Figure 2:**
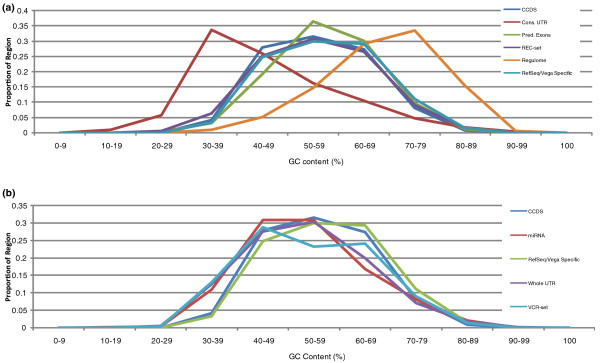
**GC content distributions for capture subregions**. **(a) **REC-set capture and **(b) **VCR-set capture.

In order to expand the regions that can be effectively targeted in capture sequence experiments, we designed two new capture reagents, termed the VCR-set and REC-set (Figure 1). The VCR-set targets the microRNA (miRNA) [21], Vega [22], CCDS, and RefSeq gene models, including predicted genes within RefSeq, with a total target size of 42 Mbp. To evaluate its ability to capture non-conserved UTRs, this design includes 8 Mbp of randomly selected UTR exons (see Materials and methods). Our second capture design, the REC-set (regulome, exons, conserved elements) aims to capture a total genomic region of 52 Mbp. In addition to the CCDS, RefSeq and Vega exons, the REC-set targets conserved UTR elements [23,24], exons that have been predicted computationally [25,26], as well as the regulome, that is, regions believed to be involved in transcription factor-mediated gene regulation [27-29]. These targeted regions represent a wide range of GC contents (Figure 2).

The reagents developed here permitted us to determine the relative 'capture ability' of subregions of the genome, compared to the CCDS. This measure can be conflated by biases introduced through the sequencing platform and alignment algorithms used. To assess the specific effect of capture on enrichment, we compared the levels of sequence coverage over specific genomic loci to that of non-enriched, WGS at the same loci.

## Results

In total, we aligned more than 54 Gbp of capture sequence data derived from seven separate libraries and five DNA samples to the human reference genome. The data were generated using both VCR-set and REC-set capture designs, and SOLiD (single-end) and Illumina (paired-end) sequencing platforms.

For the REC-set design, two libraries were constructed from DNA samples obtained from human blood, from individuals of Hispanic ethnicity (L721, L722) for Illumina sequencing. One SOLiD library was constructed utilizing DNA from a HapMap cell-line (NA12812). The VCR-set design was used to capture fragments of DNA derived from two human blood samples from individuals of European ethnicity (C45, C6) followed by Illumina sequencing, and two replicate libraries from the HapMap cell line DNA (NA12812) followed by SOLiD sequencing.

We had previously reported that sequence data from the Illumina platform routinely revealed a higher overall sequence capture enrichment yield than that found when using the SOLiD platform [6]. This was attributed to the relative ease of generating 'paired end' reads on the Illumina platform and the efficiency with which they could subsequently be precisely mapped to the genome, as well as differences in ligation strategies employed during library construction (Supplementary Table 1 in Additional file 1). Subsequent improvements in the library construction protocols for SOLiD capture sequencing have reduced the difference in the overall sequence capture enrichment yields between platforms (data not shown) [6]. To ensure meaningful comparison of data generated on the different sequencing platforms, we routinely ensured that the same number of targeted bases were covered at ≥10× for each experiment.

### Capture efficiency and coverage

**Figure 3:**
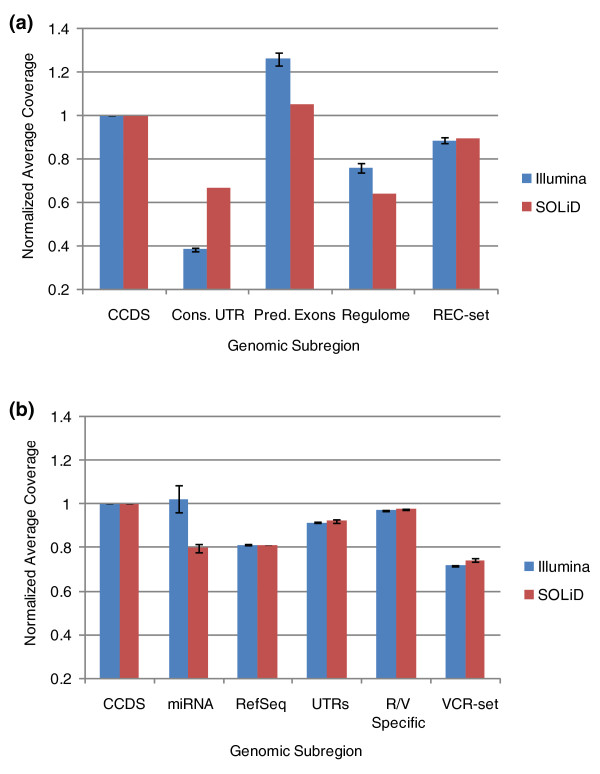
**Coverage distributions for capture subregions**. **(a) **Average coverage for subregions of REC-set design are shown as a proportion of the average coverage of the CCDS subregion. **(b) **Average coverage for subregions of VCR-set design are shown as a proportion of the average coverage of the CCDS subregion. 'R/V specific' refers to RefSeq/Vega exons not contained in the CCDS.

Genomic subregions were defined as groups of genomic segments with similar functional characteristics (UTRs, predicted exons, and so on; Figure 1). The 'capture-ability' of each subregion was defined as the average sequence coverage relative to the average coverage of the CCDS subregion. As can be seen in Figure 3a, the CCDS has approximately 10 to 15% higher average coverage than the REC-set target regions as a whole. Both the conserved UTR and regulome regions performed substantially worse than the CCDS, whereas predicted exons performed better than the CCDS. Most of the subregions in the VCR-set design performed within 10 to 20% of the CCDS (Figure 3b), the only exception being the non-conserved UTR subregion, which was substantially worse. Interestingly, the miRNA subregion performed slightly better than the CCDS when captured. For the majority of subregions, Illumina and SOLiD sequencing performed identically, with the exceptions of GC extremes (regulome, UTRs) and regions that are consistently represented by ~100 bp in the genome (for example, miRNA). Results differed by less than 1% from sample to sample (data not shown). Similar results were observed when considering the median level of coverage (Supplementary Figure 1 in Additional file 1), with the exception that the coverage performance of the REC-set and VCR-set as a whole was improved when compared to the CCDS. This is due to some CCDS targets having extremely high levels of coverage, which skews the mean coverage of these regions. Coverage differences between regions were found to be highly significant (*P *< < 0.001) by the Mann-Whitney non-parametric test. Differences in coverage seem to be driven almost entirely by GC content. CCDS exons with very high GC content had coverage that was similar to regulatory regions with high GC content, whereas CCDS exons with very low GC content had depressed coverage similar to conserved UTRs (Supplementary Figure 2 in Additional file 1).

**Figure 4:**
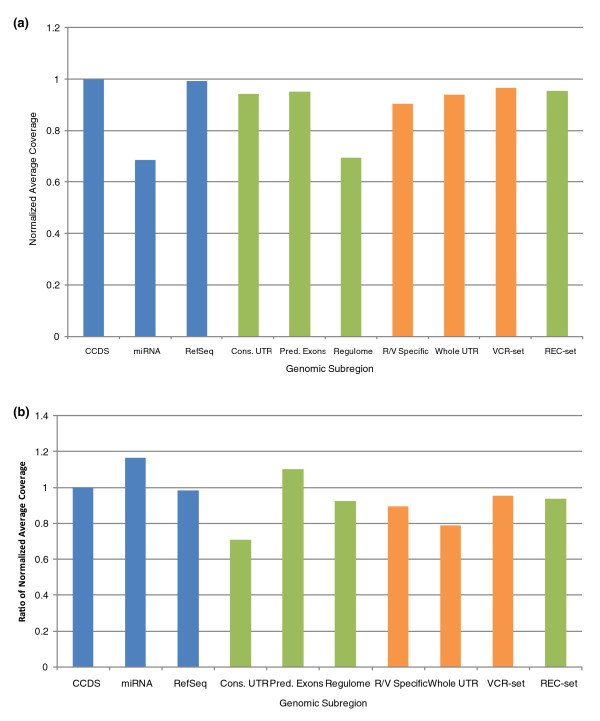
**Normalized coverage distributions**. **(a) **Coverage of genomic subregions, relative to the CCDS, after whole genome SOLiD sequencing. Green, regions specific to REC-set; orange, regions specific to VCR-set; blue, shared regions. 'R/V specific' refers to RefSeq/Vega exons not contained in the CCDS. **(b) **Proportional difference in relative coverage between capture-sequencing and WGS shows both enrichment (values > 1) and depletion (values < 1) of certain genomic subregions after capture. Green, regions specific to REC-set; orange, regions specific to VCR-set; blue shared regions.

To determine whether the observed subregional differences in capture sequence data coverage resulted from variability in the capture efficiencies, or alternatively, by biases incurred during sequencing and alignment, we compared the capture SOLiD sequencing data to their equivalent regions in SOLiD WGS data [30]. First, we determined that no particular subregion was especially prone to mismapping by artificially generating reads from these regions and mapping them back to the genome and by comparing the mapping scores (a measure of the ratio of the best to the second best alignment score) of real data aligned to each subregion (Supplementary Tables 2 and 3 in Additional file 1) and found that regions with very high variant densities (regulome, predicted exons) had mapping scores and mappabilities that were very similar to regions with the lowest variant densities (conserved UTRs); we conclude from this that the high observed variant densities in the predicted exons and regulome are likely not due to mismapping of reads. In general, the relative coverage patterns observed in WGS were similar to those from captured material (Figure 4a); however, two regions (miRNA and predicted exons) performed better than expected when captured (a positive value in Figure 4b), whereas both UTR regions performed substantially worse than WGS. These data show that some biases in recovery of sequence data from some genomic regions were incurred during sequencing, and not during the earlier capture phase.

### Variant density in subregions

We examined the density of SNV sites in different capture subregions. All data were filtered to retain reads with high mapping qualities and regions with 10× or higher sequence read coverage (see Materials and methods; Supplementary Table 3 in Additional file 1). There were differences in the discovery rate with different platforms (Illumina approximately 1/1,500 bp in the CCDS exome versus approximately 1/1,700 bp for SOLiD; Supplementary Table 4a, b in Additional file 1). Both values were similar to the variant densities observed in other exon studies (for example, Thousand Genomes Pilot Three [17]), but were considerably lower than those previously reported for the whole genome (approximately 1/1,000 bp) [31-33].

**Figure 5:**
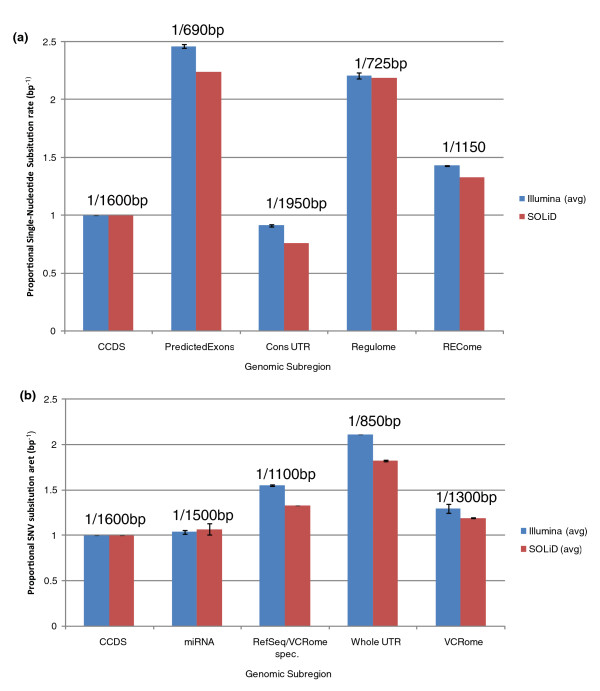
**Single nucleotide variant densities**. **(a) **Number of SNV substitutions per base pair, of REC-set subregions, as a proportion of the SNV rate of the CCDS subregion. The absolute average value from SOLiD and Illumina sequencing is given above the data point. **(b) **Number of SNV substitutions per base pair, of VCR-set subregions, as a proportion of the SNV rate of the CCDS subregion. The absolute average value from SOLiD and Illumina sequencing is given above the data point. 'R/V specific' refers to RefSeq/Vega exons not contained in the CCDS.

The evolutionarily conserved UTR portion of the REC-set design harbored 10 to 25% fewer (Figure 5a) variants (1/2,300 bp SOLiD, 1/1,625 bp Illumina) than the CCDS exome, in stark contrast to the non-conserved UTR portion of the VCR-set design (Figure 5b; Supplementary Table 4b in Additional file 1), which showed an 80 to 100% increase (1/925 bp SOLiD, 1/750 bp Illumina). Thus, there was an approximately 2.5-fold differential in variant density between conserved and non-conserved regions of the UTR.

Surprisingly, the predicted exons and regulatory regions in the REC-set design exhibited more than two times the variant density observed in the CCDS exome, a value higher than the average rate of the whole genome (1/600 bp to 1/800 bp). This suggested that these regions were either more tolerant to variation, or that these regions have increased mutation rates compared to the whole genome. Increasing or decreasing the stringency of the variant calling parameters had little effect on either the absolute variant density or the density relative to the CCDS exome (data not shown).

To confirm that this observation was not an artifact of allele-bias during the capture process, we compared these results to those obtained from two WGS SOLiD data sets [30,31] and filtered variants for predicted exons and CCDS target regions. As expected, the African genome that was previously sequenced showed a higher variant density throughout the genome (1/900 bp) than the Caucasian genome (1/1,061 bp) as well as in each subregion examined here (Table 4c in Additional file 1). The relative variant densities, however, when normalized to the CCDS exome, were approximately the same in both genomes. The observed high density of variants in the predicted exons was even more pronounced in these data, with predicted exon regions (1/714 bp) having approximately 2.5× the mutation rate of the CCDS (1/1,808 bp), and a 30 to 40% increase over the genome as a whole. To ensure that these results were not an artifact of high-throughput sequencing, we examined the variant density of these regions in HuRef [33]. The variant density in the CCDS region of HuRef was slightly depressed compared to the other two WGS datasets, but the relative variant density for each region was similar or more pronounced (Supplementary Table 4c in Additional file 1).

We examined the mutation spectrum of the observed variants in different subregions (Additional file 2). Interestingly, we found the transition:transversion ratio in the CCDS region to be 3:1, in both capture and WGS datasets, whereas across the whole genome the rate was approximately 2:1 [34]. This value was also higher than that seen in the regulome of 1.6:1. The mutation spectrum was significantly different for the regulome compared to the CCDS exome; the number of C→T and G→A mutations, as a proportion of the total number of mutations, was significantly repressed in the regulome compared to the CCDS, despite having a higher GC content and a higher proportion of CpG dinucleotides, which are known to be prone to mutation [35]. Interestingly, predicted exon subregions showed intermediate levels of all mutation types when compared to the CCDS and regulome regions. This implies that the mutation spectrum alone cannot account for the observed variant density.

Lastly, we hypothesized that predicted exons may have variant density properties identical to that of introns. Introns are thought to have a higher variant density than the whole genome because they are frequently transcribed [36]. Because no specifically intronic regions were captured by our designs, we used the WGS data and found that the intronic variant density (approximately 1/850) is slightly higher than that of the whole genome (approximately 1/1,000), but still significantly lower (*P*-value of approximately 0.0001) than that of predicted exons (approximately 1/700) (Table 4c in Additional file 1). It remains possible that the GC content of the predicted exons makes the variant density higher than that seen in the remainder of the intron.

**Figure 6:**
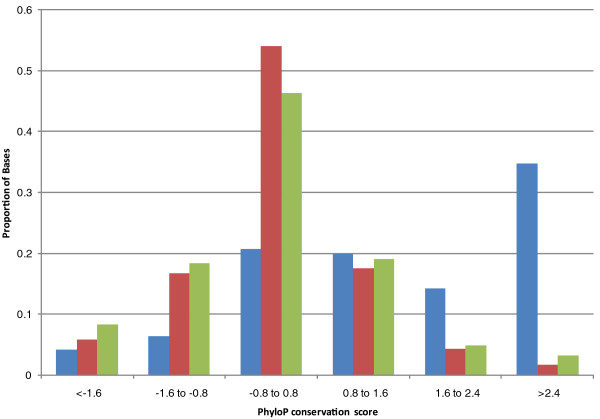
**Distribution of phyloP scores across the CCDS (blue), intronic (red) and predicted exons (green)**.

**Figure 7:**
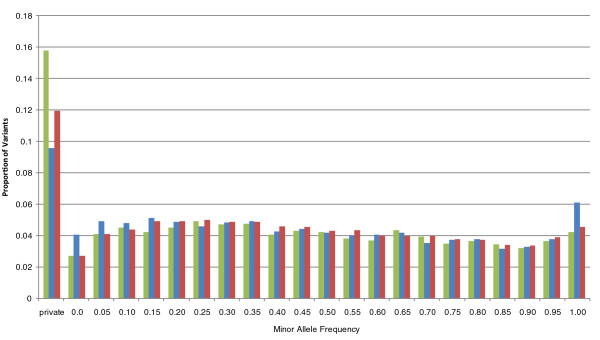
**Minor allele frequency distributions for variants in HuRef subregions: predicted exons (green), CCDS exons (blue) and introns (red)**. 'Private' indicates the variant was not found in the Thousand Genomes Project.

The high variant density we observed in the predicted exons led us to examine the evolutionary conservation of these regions relative to the introns and CCDS exons. As expected, the CCDS exons were highly conserved relative to the intronic regions. Although the predicted exons mimicked the intronic regions by having a large proportion of bases with neutral evolution scores, these regions had more bases with both high and low conservation when compared to the introns (Figure 6). We next examined the minor allele frequency distribution, using data from the Thousand Genomes Project [17], of variants in the intronic, CCDS exome and predicted exon regions for HuRef (Figure 7). Minor allele frequency distributions for CCDS and predicted exons in capture data were similar (data not shown). Although 12% of the intronic variants and only 9% of the CCDS variants were private (unseen in public databases), fully approximately 16% of the predicted exon variants were not found in data from the Thousand Genomes Project. This situation was reversed for fixed variants, with predicted exons having the smallest proportion (approximately 4%) compared to CCDS variants (approximately 6%).

## Discussion

In this study we show the efficacy of DNA capture sequencing and interrogation of variants in biologically important loci outside of the CCDS exome. These regions almost uniformly demonstrated decreased capture ability, as measured by average target coverage, when compared to the CCDS regions. Overall, both Illumina and SOLiD sequencing platforms showed similar biases in coverage of genomic subregions when measured relative to the CCDS. Importantly, capture ability appeared to be confounded by biases introduced by the sequencing technology and correlated with GC content of the target sequence, a known factor in short-read sequencing [37,38]. Particularly, conserved UTR regions, which are approximately 30% GC, and regulatory regions, which are approximately 70% GC, had approximately half of the sequence depth of coverage as the CCDS regions, approximately 50% GC. When compared to WGS (non-capture) data the same general biases were evident. However, the act of capturing the targeted regions seems to exacerbate the coverage bias by an additional 5 to 10%. The exceptions to this are the predicted exons and microRNA, where the coverage was higher than expected and the UTR regions where the coverage was as much as 25% lower than expected from the WGS data. This effect may be due to steric hindrance of probe-target binding introduced by secondary structure present in the UTR regions. These results imply that naively capturing biologically relevant loci other than the CCDS will require 20 to 40% more sequencing data to be generated than expected from the CCDS. It may be possible, however, to alter the capture reagent, perhaps by increasing the representation of some probes, in order to compensate for the empirically measured coverage biases and thus help normalize the coverage when capturing CCDS and other elements.

To our knowledge, this is the first targeted-sequence capture study of a genome-wide, diverse set of biologically important elements, allowing the investigation of variant densities in functionally relevant loci that have been hitherto undetected at a fraction of the cost of whole genome sequencing. Using both Illumina and SOLiD sequencing, we demonstrate the ability to find variants across a significantly larger target region than the CCDS. As capture sequencing enables high levels of sequence coverage, we were able to discover rare (private) variants in each sample, using similar amounts of data to that used by low-coverage, whole-genome techniques that are better suited for common variant discovery.

Illumina sequencing consistently showed higher variant densities than SOLiD sequencing. This discrepancy is likely due to differences in variant filtering parameters used for the two different sequencing types. However, it may also reflect the inherently higher accuracy of SOLiD sequencing [37]. Importantly, when measured relative to the CCDS variant density, different subregions showed remarkably similar variant densities for both sequencing platforms. Variant densities, however, were found to vary in different subregions of the genome, likely due to evolutionary conservation and base composition of these regions. The evolutionarily conserved CCDS exome and UTR regions showed variant densities of 1/1,600 to 1/1,850 bp, considerably less than the whole genome rate of 1/1,000 bp, which presumably reflects the result of purifying selection acting to remove deleterious variants. Exons specific to RefSeq, which are not in the CCDS, showed intermediate levels of variant density, 1/1,200 bp. This is likely because these loci are less essential to the organism, and mutations in these regions are less likely to be deleterious. Unlike the coding regions, the regulome showed a variant density higher than the whole genome. While this is likely due to the GC content of the regulome, we found that C→T and G→A mutations were underrepresented as a portion of all variants when compared to the CCDS. This is significant because 5-methyl-cytosine bases in CpG dinucleotides, which are over-represented in regulatory regions, are prone to spontaneous deamination to uracil and subsequent repair to thymine [39]. This would indicate there is strong selective pressure to maintain cytosine and guanine representation in the regulome compared to the CCDS exome.

Of all the regions interrogated, the predicted exons showed the highest variant density, 1/660 bp. Although these exons have a higher GC content than the CCDS, it is considerably lower than the regulome, indicating that the increased mutability of GC-rich sequence content cannot fully account for the variant density. However, we observed that the intronic variant density in WGS studies was also considerably higher than that of the whole genome. It has been reported that transcribed regions have higher variant densities than non-transcribed regions [40,41] and we surmise, therefore, that the observed variant density is a combination of these regions being actively transcribed and their high GC content. As expected from the high variant density, predicted exon regions showed a slightly higher proportion of bases with faster than neutral evolution rates than when compared to intronic regions. Unexpectedly, predicted exons also showed a slightly higher proportion of conserved bases when compared to intronic regions.

The 'exonization' of intronic elements is well documented [42-45] and computationally predicted exons have been detected in mature mRNA from RNA-seq experiments [46]. In this work we interrogated predicted exons that are flanked by canonical splice-sites and exist within known CCDS genes and thus are good candidates for inclusion in mature RNA and subsequent translation. Exons are thought to be protected from mutation [47] and the higher mutation rates in predicted-exons may then be a source of evolutionary diversity.

## Conclusions

This work has important implications for the large number of CCDS-based exome-capture experiments currently being reported. Specifically, caution should be used when extrapolating CCDS results to the entire human exome. Regions outside of the CCDS are more difficult to sequence, map and capture and require more raw sequence data than otherwise expected. Further, studies that seek to characterize human coding variation across a large number of individuals should use a diverse set of gene models to better measure and understand variation in less conserved coding elements. Consideration of non-CCDS regions in general will complicate sequence-based genome-wide disease studies due to the wider range of variant densities they exhibit, and will necessitate innovative bioinformatic data filtering strategies.

## Materials and methods

### DNA

DNA was obtained from the Corriel biorepository (catalog id GM12812). DNA was obtained from individuals under written informed consent for participation in the study. The study was approved by the institutional review board at Baylor College of Medicine and was conducted in accordance with the Helsinki declaration.

### Target regions and probe design

All annotations, except for miRNA, were downloaded from UCSC Genome Browser [48] (hg18) on 1 October 2009. Coding regions: exons for the CCDS, RefSeq and Vega gene sets were all obtained in their entirety and non-coding regions were removed internally. Conserved UTRs: UTR regions were selected from the RefSeq and Vega gene sets. A region was considered conserved if it had an LOD score ≥100 as determined by the phastCons [24] package using 17 vertebrate genomes (17-way most conserved track). miRNA: these annotations were obtained from miRNA base v13. Predicted exons: these annotations were obtained from Contrast and GenScan. Only predicted exons that occurred within the introns of known genes were used. Regulatory regions: these regions were obtained from the ORegAnno track and the Hudson Alpha transcription factor binding site (CHiP-seq) tracks. Only ORegAnno annotations that were < 50 bp were considered so as to remove non-transcription factor binding site regulatory regions and limit the total target size. CHiP-seq sites were only considered if they had an enrichment score of 300 or greater. Solution capture probes were designed and produced by Roche NimbleGen (Madison WI, USA) as previously described [6]. Nonconserved UTR: approximately 1,500 exons were randomly selected from 5' and 3' UTRs of Vega genes without any consideration for their conservation; however, they were always either the first or last exon in the annotated gene. RefSeq/Vega-specific regions: these regions are derived by algorithmically subtracting the CCDS regions from the combined Vega/RefSeq regions. For the purposes of this paper, derived regions < 50 bp were not considered as small regions, and regions at the edges of targets show lower coverage, generally.

Target regions are available as BED files in Additional files 3 and 4.

### Library preparation

Precapture libraries for SOLiD (2 μg) were hybridized in solution according to the manufacturer's protocol with minor revisions. Specifically, hybridization enhancing oligos TrTA-A and SOLiD-B replaced oligos PE-HE1 and PE-HE2 and post-capture ligation-mediated PCR was performed using 12 cycles. Capture libraries were quantified using PicoGreen (catalog number P7589) and their size distribution analyzed using the Agilent Bioanalyzer 2100 DNA Chip 7500 (catalog number 5067-1506). Capture efficiency was evaluated by performing a quantitative PCR-based SYBR Green assay (Applied Biosystems, Foster City CA, USA Inc.; catalog number 4368708) with built-in controls (RUNX2, PRKG1, SMG1, and NLK). Capture library enrichment was estimated at seven to nine cycles over background by quantitative PCR. Captured libraries were further processed for sequencing, with approximately 6 to 12 Gbs of sequence generated per capture library on either SOLiD V3 or V4 instruments (Applied Biosystems, Inc.). A complete capture protocol can be found on the Baylor Human Genome Website [49]. Illumina library preparation was conducted as previously described [6].

### Sequence data generation, alignment and variant calling

SOLiD data were aligned to the human genome (hg18) with BFast [50] and Illumina data with BWA [51]. Variants were filtered for quality as previously described [6]. Briefly, read qualities were recalibrated with GATK and a minimum quality score of 30 was required; also, the variant must have been present in at least 15% of the reads that cover the position. In addition, prior to variant calling reads with low (< 11) mapping qualities (a value based on the ratio of the best alignment score to the second best alignment score) were removed. This typically eliminates approximately 5 to 10% of the aligned reads. Sequence data were produced from either SOLiDv3 or Illumina GAII sequencing machines and are available from the Sequence Read Archive [52] with accession [SRP004501.1].

## Abbreviations

bp: base pair; CCDS: consensus coding DNA sequence; Gbp: Giga-base pair; Mbp: Mega-base pair; miRNA: microRNA; SNV: single nucleotide variant; UTR: untranslated region; WGS: whole genome sequencing.

## Competing interests

The authors declare that they have no competing interests.

## Authors' contributions

MNB aided in experiment design, analysis and manuscript preparation. MW, YQW and DM conducted capture hybridization and sequencing. JLJ provided DNA samples and helped in data interpretation. DLB, TA and RAG participated in experimental design, capture design and drafting the manuscript. All authors have approved the final manuscript.

## Supplementary Material

Additional file 1 **Supplementary data and statistics**. Experimental design, capture statistics, regional description statistics, as well as whole genome statistics. Click here for file

Additional file 2 **Mutation spectrum**. The mutation spectrum, transition:transversion ratio of discovered variants ordered by subregion. Click here for file

Additional file 3 **REC-set targets**. Targeted regions in the REC-set. Click here for file

Additional file 4 **VCR-set targets**. Targeted regions in the VCR-set. Click here for file
