# Supplementary material for: Targeted enrichment beyond the consensus coding DNA sequence exome reveals exons with higher variant densities
Source: Genome Biol. 2011 Jul 25;12(7):R68. doi: 10.1186/gb-2011-12-7-r68 (PMC3218830; doi:10.1186/gb-2011-12-7-r68)
Supplement: Additional file 1 — Supplementary data and statistics. Experimental design, capture statistics, regional description statistics, as well as whole genome statistics. [file gb-2011-12-7-r68-S1.DOCX]

**Supplementary Table 1.** Capture Statistic for REC-set and VCR-set capture-design libraries.

| **Capture Design** | **REC-set** | | |
| --- | --- | --- | --- |
| **Sequencing Type** | Illumina | Illumina | SOLiD |
| **Read Type** | 75PE | 75PE | 50Frag |
| **Sample** | L721 | L722 | NA12812 |
| **Total Reads Aligned (M)** | 73.06 | 99.78 | 200.70 |
| **Total bp Aligned (Mbp)** | 5.48 | 7.48 | 10.03 |
| **Duplicate Reads:** | 19.95% | 25.00% | 33.78% |
| **Aligned Reads On-Target After Duplicate Removal (M)** | 44.9 | 56.6 | 33.0 |
| **Percent Reads On-Target After Duplicate Removal** | 61.45% | 56.72% | 16.51% |
| **Average Coverage** | 66 | 88 | 35 |
| **Median Coverage** | 37 | 48 | 33 |
| **Targets Hit:** | 98.41% | 98.46% | 98.34% |
| **Bases with 1x+ coverage** | 96.59% | 96.92% | 94.96% |
| **Bases with 10x+ coverage** | 79.92% | 83.07% | 80.74% |
| **Bases with 20x+ coverage** | 66.43% | 71.65% | 67.38% |

| **Capture Design** | **VCR-set** | | | |
| --- | --- | --- | --- | --- |
| **Sequencing Type** | Illumina | Illumina | SOLiD | SOLiD |
| **Read Type** | 75PE | 75PE | 50Frag | 50Frag |
| **Sample** | C45 | C6 | NA12812 | NA12812 |
| **Total Reads Aligned (M)** | 68.87 | 85.59 | 193.99 | 212.91 |
| **Total bp Aligned (Mbp)** | 5.17 | 6.42 | 9.70 | 10.65 |
| **Duplicate Reads:** | 52.99% | 33.66% | 34.45% | 37.24% |
| **Aligned Reads On-Target After Duplicate Removal (M)** | 20.9 | 40.9 | 27.3 | 29.8 |
| **Percent Reads On-Target After Duplicate Removal** | 30.40% | 47.79% | 14.08% | 14.06% |
| **Average Coverage** | 68 | 94 | 39 | 44 |
| **Median Coverage** | 52 | 73 | 37 | 45 |
| **Targets Hit:** | 97.36% | 97.91% | 98.07% | 98.35% |
| **Bases with 1x+ coverage** | 95.07% | 96.44% | 93.65% | 94.85% |
| **Bases with 10x+ coverage** | 84.00% | 90.65% | 80.20% | 83.84% |
| **Bases with 20x+ coverage** | 74.89% | 84.74% | 69.49% | 75.05% |

**Supplementary Table 2**: The percentage of all possible, plus stranded, 50mers which correctly aligned back to the genomic coordinate from which they were derived for each subregion. Blue, region shared by both VCR-set and REC-set. Orange, VCR-set only regions. Green, REC-set only regions

| **Region** | **Mappability** |
| --- | --- |
| CCDS | 97.70% |
| Predicted Exons | 94.30% |
| Regulome | 95.40% |
| Conserved UTR | 94.70% |
| Whole UTR | 92.20% |
| VCR-set (whole) | 96.70% |
| REC-set (whole) | 94.60% |

**Supplementary Table 3:** Average and standard deviation mapping quality (MQ) and for Illumina sequence reads aligned to each sub region. Blue, region shared by both VCR-set and REC-set. Orange, VCR-set only regions. Green, REC-set only regions.

| **Region** | **MQ (avg)** | **MQ StdDev** |
| --- | --- | --- |
| CCDS | 55.7 | 5.6 |
| Predicted Exons | 54.8 | 7.9 |
| Conserved UTR | 55.7 | 6.3 |
| Regulome | 54.2 | 7.5 |
| Whole UTR | 55.6 | 5.1 |
| VCR-set | 54.8 | 6.0 |
| REC-set | 55.6 | 6.8 |

**Supplementary Table 4a**. Coverage and variant counts for REC-set design libraries.

| **REC-set** | **Region** | **# bp >10x coverage** | **Total Variants** | **Var. Freq.** | **Var/bp (Normalized to CCDS )** |
| --- | --- | --- | --- | --- | --- |
| **Illumina 721** | **CCDS** | 24411435 | 16439 | 1484.97 | 1.00 |
|  | **Cons UTR** | 2379258 | 1485 | 1602.19 | 0.93 |
|  | **Predicted Exons** | 6746792 | 11219 | 601.37 | 2.47 |
|  | **Regulome** | 1678861 | 2492 | 673.70 | 2.20 |
|  | **REC-set** | 46062201 | 44424 | 1036.88 | 1.43 |
| **Illumina 722** | **CCDS** | 25105820 | 16978 | 1478.73 | 1.00 |
|  | **Cons UTR** | 2702825 | 1645 | 1643.05 | 0.90 |
|  | **Predicted Exons** | 6804117 | 11303 | 601.97 | 2.46 |
|  | **Regulome** | 1760814 | 2632 | 669.00 | 2.21 |
|  | **REC-set** | 47877605 | 46257 | 1035.03 | 1.43 |
| **SOLiD (0440)** | **CCDS** | 24006540 | 13986 | 1716.47 | 1.00 |
|  | **Cons UTR** | 3367427 | 1486 | 2266.10 | 0.76 |
|  | **Predicted Exons** | 6371265 | 8310 | 766.70 | 2.24 |
|  | **Regulome** | 1489462 | 1899 | 784.34 | 2.19 |
|  | **REC-set** | 46531863 | 36056 | 1290.54 | 1.33 |
| **AVG** | **CCDS** |  |  | 1560.06 | 1.00 |
|  | **Cons UTR** |  |  | 1837.12 | 0.86 |
|  | **Predicted Exons** | |  | 656.68 | 2.39 |
|  | **Regulome** |  |  | 709.01 | 2.20 |
|  | **REC-set** |  |  | 1120.82 | 1.40 |

**Supplementary Table 4b**. Coverage and variant counts for VCR-set design libraries.

| **VCR-set Library** | **Region** | **# bp >10x coverage** | **Total Variants** | **Var. Freq.** | **Var/bp (Normalized to CCDS )** |
| --- | --- | --- | --- | --- | --- |
| **Illumina C45** | **CCDS** | 24267583 | 15275 | 1588.71 | 1.00 |
|  | **miRNA** | 54590 | 35 | 1559.71 | 1.02 |
|  | **VCR-set** | 37089976 | 30284 | 1224.74 | 1.30 |
|  | **R/V Specific** | 4793430 | 4583 | 1045.92 | 1.52 |
|  | **Whole UTR** | 5605769 | 7482 | 749.23 | 2.12 |
| **Illumina C6** | **CCDS** | 26121376 | 17094 | 1528.10 | 1.00 |
|  | **miRNA** | 58067 | 40 | 1451.68 | 1.05 |
|  | **VCR-set** | 40029048 | 33891 | 1181.11 | 1.29 |
|  | **R/V Specific** | 5284674 | 5493 | 962.07 | 1.59 |
|  | **Whole UTR** | 6031754 | 8296 | 727.07 | 2.10 |
| **SOLiD 0079** | **CCDS** | 23335741 | 13700 | 1703.34 | 1.00 |
|  | **miRNA** | 50584 | 33 | 1532.85 | 1.11 |
|  | **VCR-set** | 35413385 | 24678 | 1435.02 | 1.19 |
|  | **R/V Specific** | 4586987 | 3579 | 1281.64 | 1.33 |
|  | **Whole UTR** | 5223722 | 5550 | 941.21 | 1.81 |
| **SOLiD 0443** | **CCDS** | 24307261 | 14726 | 1650.64 | 1.00 |
|  | **miRNA** | 53177 | 33 | 1611.42 | 1.02 |
|  | **VCR-set** | 37020315 | 26792 | 1381.77 | 1.19 |
|  | **R/V Specific** | 4824483 | 3861 | 1249.54 | 1.32 |
|  | **Whole UTR** | 5520311 | 6142 | 898.78 | 1.84 |
| **AVG** | **CCDS** |  |  | 1617.70 | 1.00 |
|  | **miRNA** |  |  | 1538.92 | 1.05 |
|  | **VCR-set** |  |  | 1305.66 | 1.24 |
|  | **R/V Specific** | |  | 1134.79 | 1.44 |
|  | **Whole UTR** | |  | 829.07 | 1.97 |

**Supplementary Table 4c**. Coverage and variant counts for African[1], European[2] and HuRef[3] WGS.

| **WGS** |  | **Callable Region (bp)** | **Variants** | **Genomic**  **Basepairs/Variant** | **Normalized Variant Density** |
| --- | --- | --- | --- | --- | --- |
| **African** | CCDS | 32905260 | 21986 | 1496.65 | 1.00 |
|  | Predicted Exons | 7385826 | 12798 | 577.11 | 2.59 |
|  | Regulome | 2285641 | 2614 | 874.38 | 1.71 |
|  | Whole Genome | 3.30E+09 | 3699673 | 891.97 | 1.68 |
|  | ucsc introns | 1.23E+09 | 1744591 | 706.93 | 2.12 |
| **European** | CCDS | 32905260 | 18194 | 1808.58 | 1.00 |
|  | Predicted Exons | 7385826 | 10342 | 714.16 | 2.53 |
|  | Regulome | 2285641 | 2614 | 874.38 | 2.07 |
|  | Whole Genome | 3.30E+09 | 3110133 | 1061.05 | 1.7 |
|  | ucsc introns | 1.23E+09 | 1451568 | 849.63 | 2.13 |
| **HuRef (European)** | CCDS | 32905260 | 15363 | 2141.85 | 1.00 |
|  | Predicted Exons | 7385826 | 9392 | 786.40 | 2.72 |
|  | Regulome | 2285641 | 2267 | 1008.22 | 2.12 |
|  | Whole Genome | 3.30E+09 | 3129238 | 1054.57 | 2.03 |
|  | UCSC introns | 1.23E+09 | 1262686 | 974.11 | 2.20 |

**Supplementary Figure 1.** Median coverage of target regions for Illumina and SOLiD sequencing for REC-set and VCR-set.

**Supplementary Figure2.** Average coverage of CCDS, Regulome and conserved UTR for various levels of GC content for Illumina and SOLiD sequencing. Data omitted when <500 regions had a specified GC content.
